# Effect of Antimicrobial Stewardship on Oral Quinolone Use and Resistance Patterns over 8 Years (2013–2020)

**DOI:** 10.3390/antibiotics10111426

**Published:** 2021-11-22

**Authors:** Atsushi Uda, Katsumi Shigemura, Koichi Kitagawa, Kayo Osawa, Mari Kusuki, Yonmin Yan, Ikuko Yano, Takayuki Miyara

**Affiliations:** 1 Department of Infection Control and Prevention, Kobe University Hospital, Kobe 650-0017, Japan; katsumi@med.kobe-u.ac.jp (K.S.); hyakuta@med.kobe-u.ac.jp (M.K.); miyarat@med.kobe-u.ac.jp (T.M.); 2 Department of Pharmacy, Kobe University Hospital, Kobe 650-0017, Japan; iyano@med.kobe-u.ac.jp; 3 Division of Infectious Diseases, Department of Public Health, Graduate School of Medicine, Kobe University, Kobe 654-0142, Japan; ko1.kitgwa@gmail.com; 4 Division of Urology, Graduate School of Medicine, Kobe University, Kobe 650-0017, Japan; yym1112@gmail.com; 5 Division of Advanced Medical Science, Graduate School of Medicine, Kobe University, Technology and Innovation, Kobe 657-8501, Japan; 6 Department of Medical Technology, Kobe Tokiwa University, Kobe 653-0838, Japan; k-ohsawa@kobe-tokiwa.ac.jp; 7 Department of Clinical Laboratory, Kobe University Hospital, Kobe 650-0017, Japan

**Keywords:** antimicrobial stewardship, unnecessary antibiotic prescription, oral quinolone, bacterial resistance, educational intervention

## Abstract

Since 2014, several global and national guidelines have been introduced to address the problem of antimicrobial resistance. We conducted a campaign in a tertiary hospital to promote appropriate quinolone use through educational lectures in 2018. The aim of this retrospective study was to evaluate the changes in the following: prescription characteristics, trend of oral quinolone use, and antibiotic susceptibility of bacteria from 2013 to 2020. Antimicrobial use was assessed as days of therapy per 1000 patient-days. We found a significant reduction in unnecessary antibiotic prescriptions between December 2013 and December 2020. Significant negative trends were detected in the use of quinolones over 8 years (outpatients, coefficient = −0.15655, *p* < 0.001; inpatients, coefficient = −0.004825, *p* = 0.0016). In particular, the monthly mean use of quinolones among outpatients significantly decreased by 11% from 2013 to 2014 (*p* < 0.05) and reduced further by 31% from 2017 to 2020 (*p* < 0.001). A significant positive trend was observed in the susceptibility of *Pseudomonas aeruginosa* to levofloxacin (*p* < 0.001). These results demonstrate that the use of oral quinolones was further reduced following educational intervention and the bacterial susceptibility improved with optimal quinolone usage compared to that in 2013.

## 1. Introduction

The spread of antibiotic-resistant pathogens leads to serious health threats that affect clinical outcomes, including higher mortality rates and increased healthcare costs. A relevant review by Jim O’Neil in 2014 estimated that antimicrobial resistance could cause 10 million deaths each year by 2050 [1]. Since 2014, the World Health Organization, European Union, the United Kingdom, and the United States have submitted strategic action plans to tackle the increasing challenge of antimicrobial resistance [2,3,4]. In accordance with these guidelines, the National Action Plan on Antimicrobial Resistance was formulated in Japan in April 2016. The goals of the plan were to reduce the use of oral broad-spectrum antimicrobials, such as quinolones, cephalosporins, and macrolides (documented in 2013) by 50% by the year 2020 [5]. To address this objective, in June 2017 and December 2019, the Government of Japan’s Ministry of Health, Labor, and Welfare issued the 1st and 2nd Edition of the *Manual of* *Antimicrobial Stewardship*, respectively [6,7]. The goal of these manuals was to promote appropriate clinical management of infectious diseases as well as to reduce inappropriate and unnecessary use of antimicrobial agents without worsening patient outcomes [6,7]. To prevent the spread of antimicrobial resistance, optimal antimicrobial therapy was introduced in various hospitals.

The overuse and misuse of broad-spectrum antibiotics may lead to the development of resistant mutations and are associated with the following: antimicrobial resistance to bacteria, longer duration of hospital stay, increased medical costs, and increased risk of adverse drug reactions [8,9]. Overuse of quinolones induces resistance in bacteria through mechanisms such as specific mutations in the quinolone resistance-determining region or overexpression of efflux pumps [10]. Therefore, reductions in inappropriate and unnecessary quinolone prescriptions are required to restrict the development of resistant pathogens. However, the prescription of broad-spectrum antibiotics is more common in Japan than in Europe and the United States [5]. In particular, the use of oral quinolones in Japan increased from 2009 to 2013 [11]. Antimicrobial stewardship programs (ASPs), including educational interventions, have been associated with appropriate antibiotic use, reduced prevalence of antibiotic-resistant pathogens, and improved clinical outcomes [12,13,14]. Therefore, in addition to the issuance of several global and national ASP guidelines, we began conducting educational lectures in our hospital to promote appropriate oral quinolone usage. However, it remains unknown how antimicrobial therapy and the prevalence of resistant pathogens have changed in our hospital. The purpose of this study was to retrospectively investigate the prescription characteristics, oral quinolone use, and the antibiotic susceptibility of bacteria in our hospital from 2013 to 2020.

## 2. Results

### 2.1. Demographic Characteristics of Patients and Frequency of Oral Quinolone Prescriptions

Data regarding 676 patients who received oral quinolone prescriptions during December 2013, December 2019, and December 2020 were reviewed (outpatients, n = 404; inpatients, n = 272). The characteristics of these patients are shown in Table 1. There were no significant differences in terms of sex, age, or hospital ward (comprising both medical and surgical wards).

Table 2 shows the frequency of oral quinolone prescriptions for the patients included in this study. Oral quinolones in outpatients were more frequently prescribed for urinary tract infections (*p* = 0.006) during December 2019 than in December 2013. The following indications for oral quinolone use in outpatients were significantly lower during December 2019 and December 2020 than in December 2013: infections not requiring antibiotic prescriptions (*p* = 0.03 and *p* < 0.001, respectively) and gastroenteritis (*p* = 0.032, both). Significant reductions were observed in other infections between December 2013 and December 2019 (*p* < 0.001). Prescriptions for acute respiratory tract infections (*p* < 0.001) and dental infections (*p* = 0.015) were significantly reduced from December 2013 to December 2020. There were significant reductions in infections not requiring antibiotic prescriptions (*p* = 0.03) and acute respiratory tract infections (*p* = 0.041) between December 2019 and December 2020.

The following indications for oral quinolone use in inpatients were significantly lower during December 2019 and December 2020 than in December 2013: intra-abdominal infections (*p* = 0.034 and *p* = 0.038, respectively) and unknown infections (*p* = 0.001 and *p* = 0.003, respectively). A significant reduction was observed in other infections between December 2013 and December 2019 (*p* = 0.03). Oral quinolone prescriptions were significantly lower in inpatients during December 2020 than in December 2013 for perioperative antibiotic prophylaxis (*p* = 0.034). There was a significant increase in other infections between December 2019 and December 2020 (*p* < 0.001). No prescriptions were given to inpatients for infections not requiring antibiotic prescriptions, acute respiratory tract infections, gastroenteritis, or dental infections.

### 2.2. Antibiotic Use

**Figure 1:**
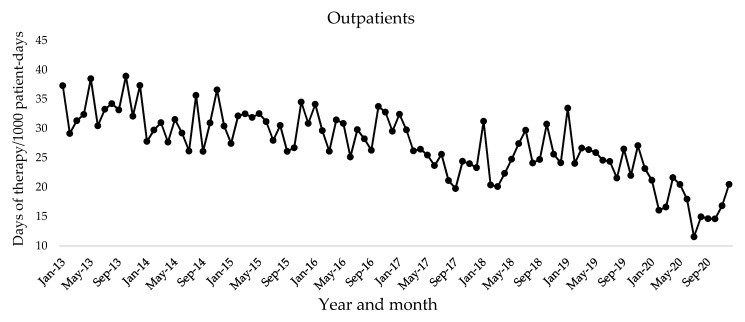
Monthly days of therapy per 1000 patient-days of oral quinolones in outpatients.

**Figure 2:**
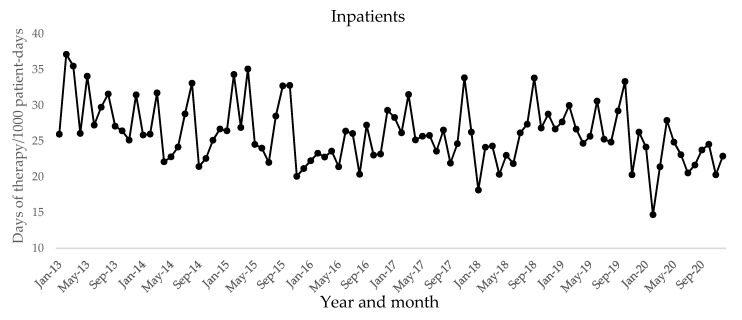
Monthly days of therapy per 1000 patient-days of oral quinolones in inpatients.

The following oral quinolones were prescribed for outpatients: ciprofloxacin, garenoxacin, sitafloxacin, ofloxacin, levofloxacin, prulifloxacin, tosufloxacin, and moxifloxacin. The prescribed oral quinolones for inpatients were as follows: ciprofloxacin, levofloxacin, garenoxacin, sitafloxacin, and moxifloxacin. We investigated the amounts of these quinolones prescribed for outpatients and inpatients among all included patients at Kobe University Hospital over 8 years. The trends of monthly days of therapy (DOTs) of oral quinolones in outpatients and inpatients are shown in Figure 1 and Figure 2, respectively. There were significant negative trends in monthly DOTs of oral quinolones in outpatients (coefficient = −0.15655, *p* < 0.001) and inpatients (coefficient = −0.004825, *p* = 0.0016) (Table 3).

The use of oral quinolones for outpatients and inpatients is shown in Table 4. The monthly mean DOTs per 1000 patient-days of oral quinolone for outpatients significantly reduced in 2014 and further decreased by 49% in 2020 compared to 2013 (2013 vs. 2020; 34.0 vs. 17.3 per 1000 patient-days, *p* < 0.001). For inpatients, there was a significant reduction of 25% in the monthly mean DOTs per 1000 patient-days of oral quinolone use from 2013 to 2020 (29.8 vs. 22.5 per 1000 patient-days, *p* < 0.001).

### 2.3. Antibiotic Resistance

The changes in the susceptibility of bacteria over the 8-year period are shown in Table 5. The susceptibility of *Pseudomonas* *aeruginosa* to levofloxacin was 84.7% in 2013, which increased to over 90% in 2015, and was maintained until 2020 (*p* < 0.001). There were no significant changes in the susceptibilities of *Escherichia* *coli* and *Klebsiella* *pneumoniae* to levofloxacin (*p* = 0.24 and 0.89, respectively).

### 2.4. Clinical Outcomes for Outpatients

The changes in the rate of outpatients administered intravenous antibiotics after oral quinolone prescriptions are shown in Table 6. There were no significant differences in the rate of patients who received intravenous antibiotic therapy (*p* = 0.71).

## 3. Discussion

In Japan, oral quinolone use increased from 2009 to 2013 [11], and in 2016, Japan’s National Action Plan on Antimicrobial Resistance was implemented to achieve a 50% reduction in the use of broad-spectrum antibiotics by 2020 using the year 2013 as baseline [5]. The purpose of this study was to retrospectively investigate the following: prescription characteristics, antimicrobial use, and microbiological data from 2013 to 2020 in our hospital. We found a significant reduction in the prescription of oral quinolones for inappropriate indications from 2013 to 2020. A significant decrease in the oral use of quinolones for outpatients was observed in 2014, with a reduction to 49% by 2020. Furthermore, there was a positive trend in the susceptibility of *P. aeruginosa* to levofloxacin. These results show that oral quinolone use was further reduced after educational intervention, and the susceptibility to *P. aeruginosa* increased with optimal quinolone usage compared to that noted in 2013.

We previously reported that educational lectures are effective in reducing the use of oral third-generation cephalosporins [15]. Educational intervention commenced in 2018, and the monthly mean DOTs per 1000 patient-days of oral quinolone for outpatients significantly decreased by 49% in 2020 compared to 2013. However, before the educational intervention, oral quinolone use had significantly decreased by 11% in 2014 and remained at a low level thereafter. Since 2014, several global and national guidelines were published to tackle the spread of antimicrobial resistance [2,3,4,5,6,7]. These guidelines may have affected changes in the prescriptions of oral quinolones. The *Manuals of* *Antimicrobial Stewardship* state that patients with acute respiratory tract infections and acute diarrhea should not be prescribed antibiotics [6,7]. In this study, to investigate the number of prescriptions for oral quinolones, representative data were collected during December 2013, December 2019, and December 2020. We found a significant reduction in unnecessary antibiotic prescriptions for outpatients. However, no prescriptions for these conditions were detected in hospitalized patients. This was because patients prescribed unnecessary antibiotics are usually mild and are treated in outpatient settings; therefore, they are not commonly treated as inpatient in hospitals. According to Asian guidelines for the prevention of surgical site infections, quinolones should be used as alternative agents for patients with a penicillin allergy [16,17]. We previously reported a reduction in the use of oral quinolones for outpatients in the Department of Oral and Maxillofacial Surgery between 2013 and 2018 after adopting the National Action Plan [18]. Consistent with these reports, we found that prescriptions of oral quinolones were significantly reduced for perioperative prophylaxis in inpatients and dental infections in outpatients. The guidelines for acute cholangitis and cholecystitis recommend that quinolones should be prescribed to patients with a beta-lactam allergy or if the isolate is susceptible to quinolones [19]. *Bacteroides fragilis* is one of the commonly isolated commensals in patients with intra-abdominal infections, and antibiotics used against anaerobic infections are often selected as empiric therapy [20]. Most oral quinolones used in Japan have no activity against anaerobes [21], and thus, oral quinolone use is considered to be inappropriate for these infections. In the present study, there was a significant reduction in the number of prescriptions for inpatients with intra-abdominal infections. These findings suggest that global and national guidelines, as well as educational interventions, may contribute to a significant downtrend in the use of oral quinolones. Although we found a considerable reduction in the use of oral quinolone for outpatients, no significant changes were detected in the number of patients who received intravenous therapy after oral quinolone prescriptions. This indicates that the reduction in quinolone prescription did not negatively influence the outcomes for these patients.

Prior use of broad-spectrum antibiotics, including quinolones, is a risk factor for acquiring quinolone-resistant *E. coli* infections, which is an independent predictor of mortality [22,23]. Switching to narrower spectrum coverage antibiotics can prevent unnecessary broad-spectrum antimicrobial use, improve patient outcomes, and be cost effective in the treatment of infectious diseases [24,25]. A retrospective observational study demonstrated that the resistance rates of *E. coli* and *P. aeruginosa* to quinolones decreased through the reduced consumption of antibiotics and surgical antibiotic prophylaxis [26]. In this study, we investigated the susceptibility of *P. aeruginosa*, *E. coli* and *K. pneumoniae* because these bacteria have high antimicrobial resistance patterns and are commonly detected as sources of community and hospital infections. The susceptibility of *P. aeruginosa* to levofloxacin significantly increased from 84.7% in 2013 to 93.3% in 2015 and remained at high levels during the study period. This improved susceptibility may be due to the reduction in unnecessary quinolone use. There were no significant changes in the susceptibility of *E. coli* and *K. pneumoniae; E. coli* is the most common bacteria identified in urinary tract infections, followed by *K. pneumoniae*. In the Japanese guidelines for clinical management of infectious diseases, oral quinolones are recommended as the first-line empiric antibiotics for these conditions [27]; a significant increase was observed in the number of oral quinolone prescriptions for outpatients in 2019. The unchanged susceptibility to levofloxacin may be related to the increase in the number of oral quinolone prescriptions for urinary tract infections in outpatients. The susceptibility of *E. coli* was low (in the 70% range) throughout the study period. In Kobe, non-extended-spectrum-β-lactamase-producing *E. coli* and *K. pneumoniae* have approximately 90% susceptibility to first-generation cephalosporins [28], and the Infectious Diseases Society of America guidelines recommend trimethoprim/sulfamethoxazole as an appropriate choice for therapy [29]. A previous study reported that restriction of oral quinolone use could lead to an increase in the susceptibility of *E. coli* [30]. To further increase susceptibility, oral quinolone use should be decreased and replaced with narrow-spectrum antibiotics.

Between December 2019 and December 2020, significant reductions were observed in the number of prescriptions for infections not requiring antibiotic prescriptions and acute respiratory tract infections. Since the beginning of 2020, we provided additional lectures directly to the representative physicians from the departments where no particular improvement was observed. This intervention could have led to the reductions in 2020; however, the world faced the threat of the coronavirus disease 2019 (COVID-19) pandemic. Many hospitals struggled with the problems posed by COVID-19, such as delaying or avoiding medical care for non-urgent cases [31]. The COVID-19 pandemic may have influenced the pattern of prescribing oral antibiotics in the year 2020.

This study had some limitations. Since it was conducted at a single institution and focused on the low incidence of non-susceptible strains to quinolones, our findings may not be generalizable. The indications for antibiotic use were collected by reviewing each patient’s medical records. We collected data over three months as representative data; hence, a measurement and sample size bias may exist. Because the study was conducted retrospectively, we could not directly assess the impact of the guidelines on physician prescribing trends. In many cases, physicians tend to shift their prescription preferences rather than using oral quinolones; however, to accurately assess the changes in oral quinolone use, we excluded the data on patients who required long-term oral quinolone prescriptions. This makes it difficult to investigate trends in the use of other antibiotics. In 2020, COVID-19 wreaked havoc in many hospital settings worldwide. Influences of COVID-19 on the prescription of antibiotics were reported [32,33]. To assess changes due to COVID-19 in the number of prescriptions, we compared the data between 2019 and 2020; however, the impact of COVID-19 on the results from 2020 could not be sufficiently investigated. Lastly, we implemented other ASPs, such as audit and feedback programs to promote appropriate intravenous antibiotic use [13,34], and educational interventions to optimize oral third-cephalosporin use during the investigation period [15,18]. Therefore, we may not have fully evaluated the effects of our educational interventions to promote appropriate oral quinolone use.

## 4. Methods

### 4.1. Setting and Patients

We conducted an observational retrospective study between 1 January 2013 and 31 December 2020 at Kobe University Hospital. We investigated patients who were prescribed oral quinolones and excluded those who required long-term oral quinolone prescriptions for diseases such as tuberculosis, non-tuberculous mycobacterial diseases, osteomyelitis, and prosthetic joint infections. Patients’ medical data were collected from electronic medical records.

### 4.2. Educational Intervention for Promoting Appropriate Antimicrobial Use

We conducted lectures for all staff in June 2018, February 2019, February 2020, and June 2020, and for representative physicians from each department in July 2018 and June 2019. The contents of the lectures included the following items: goals to reduce the use of oral broad-spectrum antimicrobials, problems of correlating antibiotic consumption and antimicrobial-resistant bacteria, typical cases of inappropriate use of oral antibiotics, and rational antimicrobial strategies, including de-escalation therapy. In departments where no particular improvement was observed, we provided additional lectures directly to the representative physicians in January–March, June, and July 2020.

### 4.3. Outcomes

To investigate the representative data of indications for oral quinolone prescriptions, we compared data for all patients during December 2013, December 2019, and December 2020. We retrospectively investigated the number of oral antibiotics administered to outpatients and inpatients. We evaluated oral quinolone prescriptions by calculating the total number of oral quinolone prescriptions normalized per 10,000 patient-days. Prescriptions of antibiotics for acute respiratory tract infections (common cold, mild acute rhinosinusitis without confirmed streptococcal pharyngitis in a rapid antigen test or throat swab culture, and acute bronchitis) and acute diarrhea were defined as infections not requiring antibiotic prescription use [6,7]. The use of oral antibiotics was evaluated based on monthly DOTs per 1000 patient-days. The outpatient numerator was calculated by dividing the total number of outpatients in each period. The susceptibility of *P. aeruginosa*, *E. coli*, and *K. pneumoniae* to levofloxacin was evaluated using microbiological laboratory records, and the first isolate was used for analysis. We investigated the characteristics of non-extended-spectrum-β-lactamase-producing bacteria. Data on the number of patients administered intravenous antibiotics were collected within 30 days after the prescription of oral quinolones.

### 4.4. Statistical Analysis

Non-parametric variables were analyzed between the groups using the Mann–Whitney U test, followed by Holm’s post hoc test. Analysis of categorical variables was conducted using the chi-square test or Fisher’s extract test with post hoc Holm adjustment. We analyzed the longitudinal trend of oral quinolone use using a simple linear regression model and evaluated the correlation between the use of antibiotics and time changes. Statistical analysis of the difference in the means of oral quinolone use was performed by one-way analysis of variance, followed by Dunnett’s post hoc test. The Cochran–Armitage test for trends was used to compare the susceptibility of bacteria and trends in the patients who received intravenous antibiotics between the groups. All statistical analyses were performed using EZR (Saitama Medical Center, Jichi Medical University, Saitama, Japan). The threshold for statistical significance was set at *p* < 0.05.

## 5. Conclusions

A significant reduction was observed in unnecessary oral quinolone prescriptions between 2013 and 2020. There was a negative trend in the use of oral quinolones for outpatients and inpatients following educational intervention. The susceptibility of *P. aeruginosa* to levofloxacin improved during the study period. These findings demonstrate that bacterial susceptibility improves with reductions in unnecessary prescriptions and the use of oral quinolones following educational interventions. More rigorous antimicrobial stewardship interventions are necessary to optimize oral quinolone use and minimize the prevalence of resistant pathogens.

## Figures and Tables

**Table 1:** Demographic characteristics of patients who received oral quinolone prescriptions.

Characteristic	December 2013	December 2019	December 2020	*p*-Value 2013 vs. 2019	*p*-Value 2013 vs. 2020	*p*-Value 2019 vs. 2020
Outpatients	n = 161	n = 128	n = 115			
Male sex, n (%)	85 (53)	81 (63)	58 (50)	0.19	0.79	0.18
Age, median years (IQR)	69 (52–79)	70 (56–77)	68 (56–76)	1	1	1
Medical ward, n (%)	72 (45)	48 (38)	39 (34)	0.53	0.28	0.65
Inpatients	n = 134	n = 77	n = 61			
Male sex, n (%)	90 (67)	52 (68)	36 (59)	1	1	1
Age, median years (IQR)	69 (56–77)	68 (57–77)	73 (62–78)	0.84	0.12	0.18
Medical ward, n (%)	66 (49)	41 (53)	31 (51)	1	1	1

IQR, interquartile range.

**Table 2:** Frequency of oral quinolone prescriptions in each indication (prescriptions/10,000 patient-days).

Indications for use	December 2013	December 2019	December 2020	*p*-Value 2013 vs. 2019	*p*-Value 2013 vs. 2020	*p*-Value 2019 vs. 2020
Outpatients						
Infections not requiring antibiotic prescriptions	6.4	2.7	0.5	0.03	<0.001	0.03
Acute respiratory tract infections	4.7	2.7	0.5	0.172	<0.001	0.041
Gastroenteritis	1.7	0	0	0.032	0.032	
Dental infections	2.5	0.7	0.2	0.097	0.015	0.625
Perioperative antibiotic prophylaxis	10.9	8.5	6.1	0.47	0.069	0.47
Surgical site infections	0.7	0.2	0.2	1	1	1
Prevention of febrile neutropenia	4.2	4	6.8	1	0.32	0.32
Otorhinolaryngology infections	2	0.2	0.5	0.09	0.19	0.97
Pneumonia	2	1.3	1.6	1	0.58	1
Skin and soft tissue infections	2	0.2	0.7	0.09	0.39	0.59
Intra-abdominal infections	3	2	2.3	1	1	1
Urinary tract infections	4.7	11	8.7	0.006	0.079	0.33
Other infections	6	0.9	3	<0.001	0.083	0.083
Unknown infections	6.2	5.4	3	0.72	0.15	0.27
Inpatients						
Perioperative antibiotic prophylaxis	8.3	7.1	2.7	0.75	0.034	0.074
Surgical site infections	0.4	0.8	1.3	1	1	1
Prevention of febrile neutropenia	24.5	21.7	17.8	0.81	0.41	0.81
Otorhinolaryngology infections	0.8	2.1	0	0.82	0.82	0.26
Pneumonia	3.2	4.6	2.7	1	1	1
Skin and soft tissue infections	1.6	1.7	0	1	0.45	0.45
Intra-abdominal infections	9.1	3.8	3.6	0.034	0.038	1
Urinary tract infections	14.2	12.5	12.9	1	1	1
Other infections	4.8	0.8	7.6	0.03	0.26	<0.001
Unknown infections	10.7	2.1	2.7	0.001	0.003	0.91

**Table 3:** Longitudinal trend of days of therapy from 2013 to 2020.

	Coef.	*p*	R-Squared
Outpatients	−0.15655	<0.001	0.5895
Inpatients	−0.004825	0.0016	0.1015

**Table 4:** Days of therapy of included patients who received oral quinolones from 2013 and 2020.

	2013	2014	2015	2016	2017	2018	2019	2020
Outpatients	34.0 (3.1)	30.2 (3.2) *	30.4 (2.6) *	29.8 (2.8) *	25.2 (3.3) ***	25.5 (3.6) ***	25.5 (3.0) ***	17.3 (3.0) ***
Inpatients	29.8 (3.9)	25.9 (3.6)	27.4 (5.1)	24.1 (2.5) **	26.6 (3.1)	25.1 (4.0) *	27.1 (3.3)	22.5 (3.1) ***

Data are expressed as mean days of therapy per 1000 patient-days per month (standard deviation). * *p* < 0.05, ** *p* < 0.01, *** *p* < 0.001 compared to 2013.

**Table 5:** Susceptibility of each bacterium to levofloxacin over an 8-year period.

Bacteria	2013	2014	2015	2016	2017	2018	2019	2020	*p*
*Pseudomonas aeruginosa*	194/229 (84.7)	233/271 (86.0)	249/267 (93.3)	253/272 (93.0)	232/250 (92.8)	249/273 (91.2)	314/334 (94.0)	244/266 (91.7)	<0.001
*Escherichia coli*	312/396 (78.8)	295/387 (76.2)	329/419 (78.5)	319/424 (75.2)	320/438 (73.1)	338/431 (78.4)	378/499 (75.8)	427/569 (75.0)	0.24
*Klebsiella pneumoniae*	177/177 (100.0)	182/188 (96.8)	170/175 (97.1)	200/201 (99.5)	237/240 (98.8)	237/242 (97.9)	262/267 (98.1)	277/280 (98.9)	0.89

Data are expressed as follows: susceptible strains/total (%).

**Table 6:** Patients administered intravenous antibiotics after oral quinolone prescriptions as outpatients.

	2013	2014	2015	2016	2017	2018	2019	2020	*p*
Rate of intravenous antibiotic therapy	258/2409 (10.7)	240/2254 (10.6)	248/2291 (10.8)	279/2445 (11.4)	260/2062 (12.6)	232/2086 (11.1)	193/2075 (9.3)	161/1486 (10.8)	0.71

Data are expressed as follows: Patients who received intravenous antibiotics/total (%).

## Data Availability

Data sharing is not applicable to this article.
